# When mathematics meets physical activity in the school-aged child: The effect of an integrated motor and cognitive approach to learning geometry

**DOI:** 10.1371/journal.pone.0196024

**Published:** 2018-08-08

**Authors:** Mladen Hraste, Andrea De Giorgio, Petra Mandić Jelaska, Johnny Padulo, Ivan Granić

**Affiliations:** 1 University of Split, Faculty of Science, Independent Department of Social and Human sciences, Split, Croatia; 2 eCampus University, Faculty of Psychology, Novedrate (CO), Italy; 3 University of Split, Faculty of Kinesiology, Split, Croatia; 4 Tunisian Research Laboratory “Sports Performance Optimization”, National Center of Medicine and Science in Sport, Tunis, Tunisia; 5 University of Split, Faculty of Electrical Engineering, Mechanical Engineering and Naval Architecture, Department of General Courses, Split, Croatia; Waseda University, JAPAN

## Abstract

Mathematics is a science which can lead to both anxiety in children and teaching difficulties in teachers. Together, these two difficulties can increase the time spent in teaching and learning mathematics. The aim of this study is to examine the efficiency of a new integrated mathematics/geometry and physical activity program, specifically structured for increasing learning in fourth-grade pupils. Thirty-six children (age 10.36±0.55) were divided into an experimental (n_1_ = 19) group and a control (n_2_ = 17) group. The experimental group of subjects learned mathematics and geometry via the integrated teaching method, while the control group of subjects learned these subjects via traditional teaching methods. We administered two ad hoc tests, before and after the intervention, in order to study its effect. One test was on geometry knowledge and the other on mathematics, in which there were questions about the implemented teaching topics: rectangles, squares and their perimeters. Using a factorial 2×2 ANOVA, the results after four weeks indicated that the group of subjects who gained their mathematics and geometry knowledge through our intervention program were significantly more successful (*P*<0.05) than the control group. Our results suggest that the integrated teaching method proposed here could be considered a useful and efficient method for teaching mathematics and geometry based on motor tasks.

## Introduction

Motor and cognitive skills influence each other from childhood through physical activity (PA) [1–3]. The theory of a close relationship between motor and cognitive development was proposed by Gesell and Thompson (the so-called Gesell’s theory), where the authors described a biological perspective assuming that motor, cognitive and physical development are all determined primarily by biological predispositions [4]. The scientific literature demonstrates that cognitive and motor development follows developmental timetables and shows particularly that it is possible to observe an accelerated progression in the kindergarten and elementary school years and a protracted development into adolescence [5]. Atypical or delayed motor development is linked with evident cognitive deficits and vice versa [6]. However, it has already been demonstrated that in cases of intellectual disability, PA, environmental enrichment and education can improve motor and cognitive performance, both in animals [7–9] and in humans [10–13].

For obvious reasons, the integration of different and heterogeneous teaching with respect to content and methods makes the educational process both dynamic and interesting and is aimed at improving the child’s ability to learn [14]. The integrated approach consists of the planning and organization of the teaching of different disciplines and is considered to be a more effective method of teaching when the new knowledge presented is related to already well-known concepts [15]. The established connection between physical education and other school subjects shows that a model of integrated teaching needs to be developed [16]. By synchronizing parts of the body and the mind, improved motor skills, coordination, memory, reading, speech, language and mathematical skills will be achieved with an improved balance, therefore reducing stress, anxiety and hyperactivity in children [17–23].

Moreover, research on the brain has spurred further research dealing with the knowledge and directing of human activity. Following the results, scientists have called for the review and possible restatement of assumptions about learning and teaching [24,25]. According to Hart’s theory [26], the most effective learning occurs when stimuli from the outside *challenge* the student’s brain to respond to them and to integrate them into a large number of existing programs, as well as expanding the existing programs or developing new programs [27–29]. To encourage learning, it is important to create an environment in which students feel relaxed and satisfied [27–29] and to use examples from everyday life [28–30]. Furthermore, many studies have shown that exercise and movement have a positive impact on the human brain. Based on scientific knowledge gathered from around the world, associations and initiatives have been developed [17,30] that advocate greater use of PA in all spheres of life, including methods of education.

Hannoford has demonstrated the importance of integration of body and mind, as well as the impact of a coherent functioning of the learning process and memorization [31]. Several studies have indicated that PA can affect academic achievement through a variety of direct and indirect physiological, cognitive and emotional mechanisms, as well as the mechanism of learning [31–33]. A well-known fact is that many schoolchildren do not like mathematics, consider it the most difficult school subject and simply wish that mathematics would *disappear* [34]. Many authors have discussed how motor development and cognitive development affect each other in childhood [35,36]. In mathematical tasks, children must focus on, and shift their attention between, dimensions of objects, (i.e., color and shape) or the precise characteristics of mathematical problems [37]. The relationship between executive functions and motor-skill competency is also confirmed with respect to intellectual disabilities, where locomotor and object control skills are impaired as well as higher-order executive functions [38,39]. Nonetheless, few authors [40] have discussed how fine motor coordination underlies the child’s overall level of cognitive and academic functioning. Fine motor coordination may be considered an essential prerequisite for other cognitive processes, such as attention or visuomotor integration, which are more directly related to mathematical achievement [41,42]. It is widely demonstrated in published research that visuomotor integration is strongly linked to children’s concurrent and longitudinal mathematical achievement [41,43]. Mathematical skills can be supported by visuomotor integration because mathematical concepts are also based on mental representations of objects. This mental representation is developed by interaction between children and physical objects in play [44]. In this way, children are able to spatially represent and interpret numerical information [45] and use adaptive strategies to solve problems [46]. Playing sports helps in developing positive characteristics such as perseverance, consistency, speed of mental reactions and faster adaptation to new situations. Exercise improves orientation in combination with spatiotemporal relations. It encourages mental activity and quickly removes mental fatigue by employing physiological functions related to movement and energy processes enhancing mental function [47]. As well as the cognitive benefits, there are other benefits of PA that affect what happens in the classroom and out of school. These include reduction of aggression [48–50], reduction of depression [51] and better health [48].

DeFrancesco and Casas have demonstrated [52] the effects of a two-week period of integrated teaching of mathematics and physical education (PE) compared with a traditional form of teaching mathematics. Fahiminezhad and colleagues [53] have shown that a three-month period of integrated teaching of mathematics and PE showed a strong positive effect compared with traditional methods. Another study shows that integrating movement across the primary mathematics syllabus is feasible and efficacious [54]. These studies take into account the fact that PE is quite different from PA. The terms are often used interchangeably, but PE contains PA because PE is based on a sequence of learning which provides learning opportunities, appropriate instruction and meaningful and challenging content for all children [55,56].

Here, we structured a PA program that can be considered something more than simple PA but is not a replacement for PE.

The aim of this study is to examine the efficiency of a new method of integrated teaching of mathematics/geometry and PA among fourth-grade pupils.

Given the defined goal of the research, it is possible to set out four basic hypotheses: i) respondents in the control and experimental groups will not be significantly different in terms of knowledge of geometry in the initial measurement, ii) respondents in the control and experimental groups will be significantly different in terms of knowledge of geometry after the experiment, iii) the control group of respondents will be significantly more successful in terms of changes in knowledge of geometry between the initial and final measurements and iv) the experimental group of respondents will be significantly more successful in terms of changes in knowledge of geometry between the initial and final measurements.

## Materials and methods

The sample of 36 pupils (fourth grade of elementary school, age 10.36±0.55) were randomly assigned to an experimental (n_1_ = 19) or a control (n_2_ = 17) group. Each group of subjects was taught mathematics by his/her teacher of elementary education. The principal of the school and the parents gave their written consent (before the investigation) for this research, which was in accordance with the fourth-grade program. The Ethics Committee of the Faculty of Kinesiology in Split gave its written permission for the implementation of the research before the investigation, in accordance with the Helsinki declaration. Two children, one from each group, were identified as Univariate outliers and were removed from the sample of respondents.

### Experimental design

**Fig 1:**
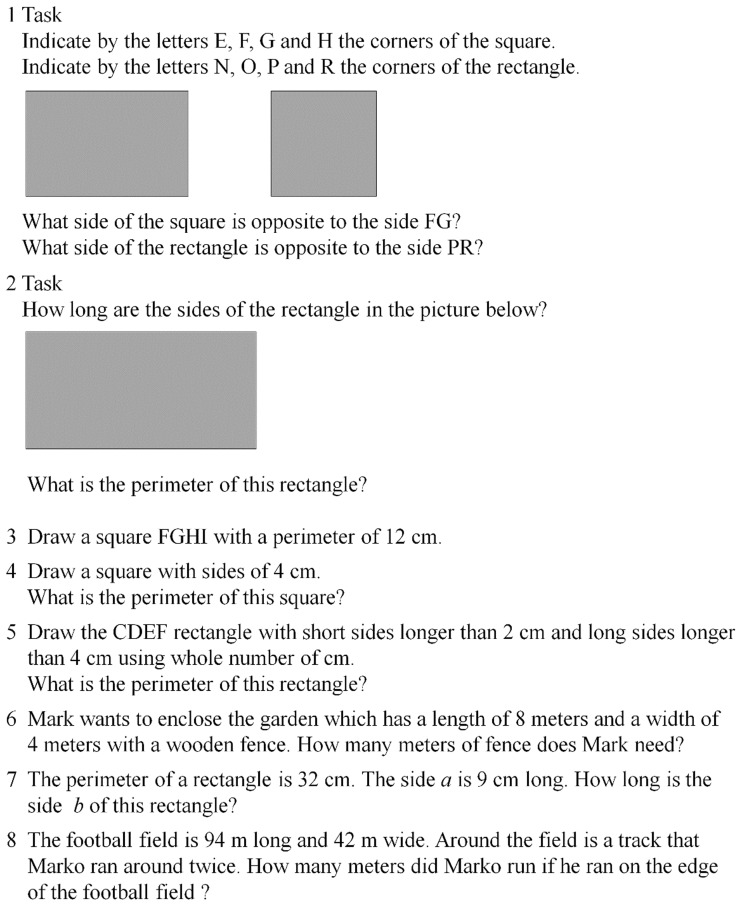
The math test.

**Fig 2:**
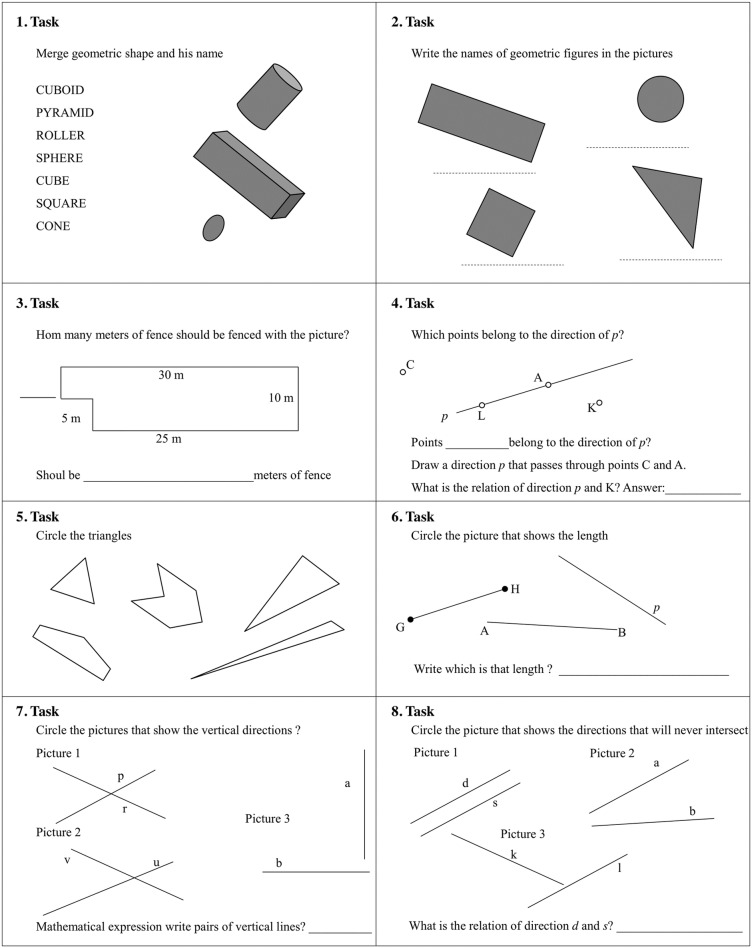
The geometry test.

This investigation was performed in March 2016 over three weeks and the experiments were always performed during the morning to avoid any circadian effects [57]. In the first and last week we performed the tests and retests (Monday and Wednesday at 9 a.m.) for the reliability of both the mathematics (Fig 1) and geometry (Fig 2) assessments, in randomized order. In the second week (Monday, Tuesday, Wednesday, Thursday), the PA was administered, always at 9 a.m., in randomized order for both experimental and control groups. Then, the two tests were administered pre- and post-intervention. In particular, the mathematics test was approved by the National School Program as a standard test for assessing mathematical knowledge. It consisted of eight tasks covering the topics of the rectangle, the square and their perimeters, gained through integrated learning methods determined by the tasks. Both tests were evaluated using a five-point Likert scale. The total number of points was the sum of the points gained in all eight assignments. It was possible to achieve a maximum of 24 points in the test. In order to standardize the procedure for both the experimental and control groups, all tests were designed under the supervision of one professor of mathematics and one professor of kinesiology.

### Testing procedures

**Fig 3:**
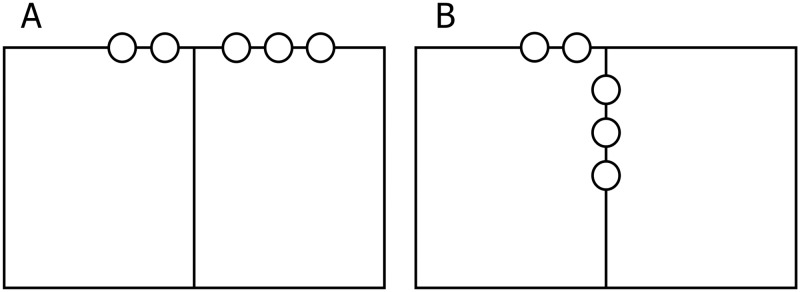
The introductory part of the lesson.

**Fig 4:**
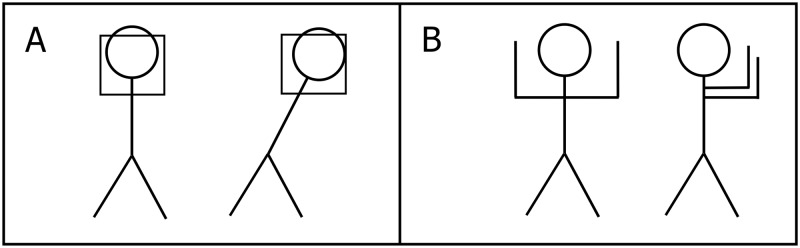
Rotating the body with the arms in the position of a right angle.

**Fig 5:**
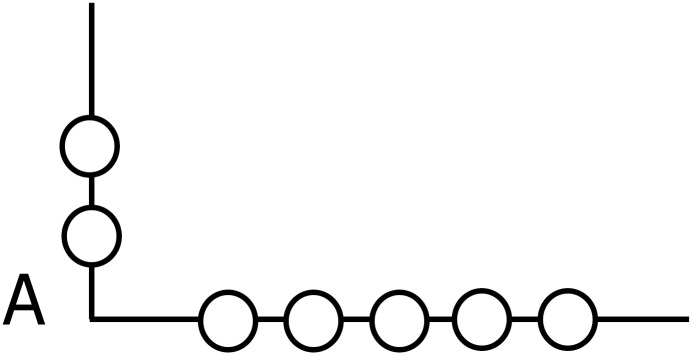
Pupils’ lesson.

**Fig 6:**
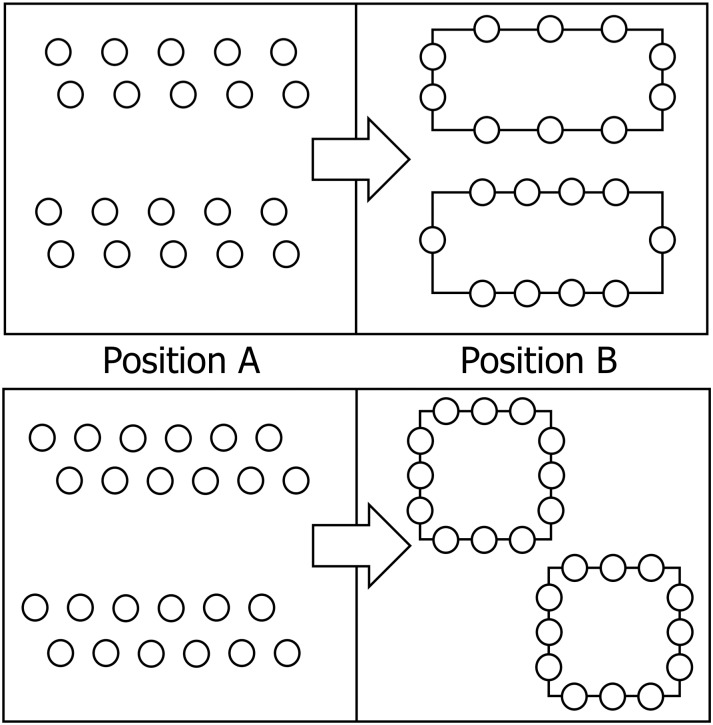
Games lesson.

**Fig 7:**
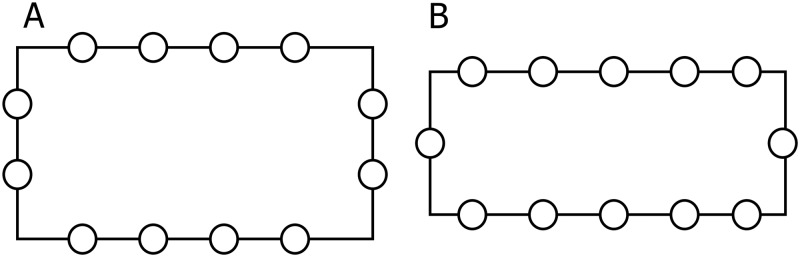
Final part of the lesson.

Initial administration of both tests was performed before the intervention. In the initial testing, both mathematical and geometrical knowledge from previous educational periods was checked. The final measurement was performed immediately after the end of the intervention. All investigations were performed in a large classroom with a temperature and relative humidity for each session ranging between 22°C and 24°C and 25% and 27% respectively. The control group of subjects learned the envisaged knowledge of the curriculum topics listed using traditional teaching methods for mathematics. The experimental group of subjects learned the envisaged knowledge of the above teaching topics exclusively through our integrated PA program. The experiment consisted of four integrated lessons of mathematics/geometry and PA. Each lesson lasted for 45 minutes and lessons took place over four weeks. According to the curriculum for the fourth grade of elementary school, teaching the topics of rectangles, squares and their perimeters is expected to be accomplished in four teaching sessions of 45 minutes each. Each lesson consisted of an introductory, a preparatory, a main and a final part. Prior to each exercise, the teacher placed all the participants in a straight line and every exercise was explained as well as clearly demonstrated. Subsequently, the pupils executed the exercises one by one. The introductory parts of the lessons (lasting four minutes each) were carried out by running, walking and/or other games assignments. The assignment was intended to get pupils moving, combining walking and running at a low to medium intensity along the edges of rectangles and squares that bounded a court with dimensions of 9×9 meters (Fig 3). The preparatory parts of the lessons (lasting seven minutes each) were carried out via six exercises in which the pupils used their arms and/or legs to form a right angle, a rectangle and/or a square (Fig 4). During the main part of the lessons (lasting 30 minutes each) pupils acquired geometrical knowledge of right angles, rectangles and squares through various games (Figs 5 and 6). For example, the pupils followed the teacher’s verbal instructions to complete the tasks: “Which group can form a right angle with the vertex at point A, using your bodies, as quickly as possible?” or “Which group can form a rectangle as quickly as possible, using your bodies, moving from area A to area B?” Pupils in area A thus formed a rectangle in space B. Note that points or areas A and B are shown for clarification in the figures, for one or two (different) destinations. The winner was the team that formed a regular rectangle first. Similarly, in the second game the students had the task of forming a square. The second game involved only squares. The aim was for students to form a square and they noticed that the square was as a geometric figure in which all four sides were equal in length. The winner was the team that formed a proper square in area B first. The final parts of the lessons (lasting four minutes each) were spent playing a game where pupils were given the task of using their bodies to form the maximum number of rectangles and/or squares from the group of 12 pupils. For example, the pupils followed the teacher’s verbal instruction: “How many different rectangles can be formed by 12 pupils?” (Fig 7).

### Statistical analysis

The reliability of the measuring instruments was verified using the test-retest method. In the retest method, the concept of parallel forms was used. To assess the reliability of the measures, an intra-class correlation coefficient (ICC) was used. For the tests, descriptive statistical parameters for each group were calculated separately: arithmetic mean (AS), standard deviation (SD), median (Med), minimum score (Min), maximum score (Max), skewness (α_3_) and kurtosis (α_4_). The normality of the distributions of all the variables was checked using the Kolmogorov-Smirnov test with the Lilliefors correction. A mixed-design 2×2 ANOVA was applied to identify the significance of the impact of the between-subjects factor *Group* (control vs. experimental) and the within-subjects factor *Treatment* (initial vs. final) on the observed measurements. Fisher’s LSD post hoc test was applied to detect particular differences. As well as the above-mentioned analysis, Bartlett’s test of homogeneity of variance was also used. Effect sizes for F-statistics were expressed as partial eta squared (η_p_^2^) values. The type one error was set as α = 0.05. For the processing of results, we used the data analysis software system Statistica v. 13.2 for Windows (Dell Inc., Tulsa, OK, USA).

## Results

The results indicate a high reliability for the tests used for estimation of geometric knowledge in the initial measurement (ICC = 0.93) and the final measurement (ICC = 0.89). Table 1 shows the central and dispersion parameters achieved in terms of knowledge of geometry for both groups in the initial and in the final measurement.

**Table 1:** Indicators of descriptive statistics for the experimental and control groups of subjects.

Geometric knowledge	M±SD	%	Med	Min/Max	α_3_	α_4_	KS-LC
**EGIM**	15.23±3.47	63.45±14.46	15.00	8.00/11.00	-0.62	0.06	>0.20
**EGFM**	18.83±3.33*	78.46±13.88	19.00	10.00/23.00	-0.61	-0.14	>0.20
**KGIM**	17.55±3.02	73.13±12.58	19.50	11.00/24.00	-0.46	-0.05	>0.20
**KGFM**	19.42±4.08	80.92±17.00	19.00	9.00/24.00	-1.03	0.92	<0.05

Note: EGIM is the experimental group initial testing, EGFM is the experimental group final testing, KGIM is the control group initial testing and KGFM is the control group final testing. M±SD is mean ± standard deviation, % is percentage of arithmetic mean with respect to successful completion of the test, Med is the median score, Min/Max is the minimum/maximum score, α_3 is the_ skewness, α_4_ is the kurtosis and KS-LC is the significance of the Kolmogorov-Smirnov test with the Lilliefors correction.

The results of the Kolmogorov-Smirnov test clearly indicate that all variables are normally distributed. The parameters α_3_ and α_4_ further confirm the normality of the distributions. In addition, using the Bartlett test, the homogeneity of variance was determined between groups as χ^2^ = 0.44, *P* = 0.61 in the initial measurement and χ^2^ = 0.39, *P* = 0.53 in the final measurement. Furthermore, the results of factorial mixed design between/within the 2×2 ANOVA show a statistically significant impact for the factor *Group* (F_1,36_ = 5.051; *P* = 0.031; ŋ_p_^2^ = 0.123), and the factor *Treatment* (F_1,36_ = 7.760; *P* = 0.008; ŋ_p_^2^ = 0.177), while the interaction *Group × Treatment* appears not to be significant (F_1,36_ = 1.766; *P* = 0.192; ŋ_p_^2^ = 0.047). The significant impact of the factor *Group* points to the differences between the two groups. However, from the post hoc analysis of interaction effects, it can be observed that there was no significant difference between the groups in the initial testing or in the final testing (*P*>0.05). This confirms the first hypothesis and leads to rejection of the second. Furthermore, the significant impact of the factor *Treatment* and additional insight into Fisher’s LSD post hoc analysis of interaction effects indicate that there is no statistically significant difference (*P*<0.05) within the control group between the initial and final measurements, which leads to rejection of the third hypothesis. A statistically significant difference within the experimental group of respondents between the initial and the final measurements can be identified, thereby confirming the fourth hypothesis.

## Discussion

We assumed that the observed differences between the initial and the final sets of measurements in the experimental group of subjects were directly influenced by the experimental program. These results indicate that the teaching of mathematics/geometry through our PA program provides a better and more efficient method of teaching than traditional methods. The data obtained in this study confirm the findings of other studies that the integrated method of teaching has a greater effect than traditional teaching methods [53,58,59].

Additionally, the results of our study are in accordance with another study which also confirmed the effectiveness of integrated teaching of mathematics and PE [54].

Kim and collaborators, in a recent interesting article [60], investigated the relationships among motor and cognitive processes and mathematical skills, demonstrating that motor development and cognitive development are already strongly and dynamically interrelated in early childhood. In particular, the authors argue that visuomotor integration and mathematics exhibited ongoing reciprocity. This study is in accordance with the previous literature [17,30] and suggests the importance of organizing teaching to include PE or a specific PA program. Moreover, the effects of motor activity on cognitive skills and the brain have also been found in studies of intellectual disabilities, both in humans and in animals [7,61], and in ADHD (one of the most common mental disorders affecting children) [62]. This demonstrates the importance of implementing PA programs during early childhood.

Our results may be in line with the scientific literature on the effects of integration of two subjects, as they are the result of teaching which is organized through the use of games. Furthermore, our study provides yet another contribution to the previous findings [33–35] pointing out that education should be integrated with physical activity. Failure to use integrated teaching will certainly lead to insufficient realization of children’s potential to improve their knowledge and skills to the maximum. The results of this study have certain limitations but can also provide guidelines for future research. One of the drawbacks is the short duration of the experimental teaching. There is a high probability that if this method of teaching lasted for a longer period of time it would provide a more complete picture of the effects of integrated teaching. In addition, the different levels of initial geometrical knowledge of the pupils tested should be taken into account. Another drawback of this research is the fact that this was the pupils’ first experience of this type of teaching. A further limitation could be identified by distinguishing instructional and teaching effects from school effects, due to the fact that there are complicated cross-level interaction effects on achievement in mathematics [63]. Another limitation lay in the fact that different teachers taught the lessons to children in the experimental and control groups. Future research should increase the number of pupils taking part in the same experiment. It would probably be better to divide the pupils according to the same criteria, allowing them to be classified into two or more equal groups. The authors believe that the effect size of this research has a certain weight, which is, however, very difficult to determine from the observed limitations of the research. Very powerful effects resulting from quarterly teaching have been recorded in a previous study [52].

### Conclusion

The results implicitly confirmed that the integrated teaching of mathematics/geometry and PA is more effective than the traditional methods prevalent in schools today. Accordingly, the results indicate the dominance of the synergic effects of cognitive and motor components of the central nervous system, compared to the cognitive component only, without taking the motor component into consideration. This research supports the claim that integrated teaching is a modern method of teaching and should be carried out more often. Considering that learning is a complete process and is not just about memorizing and thinking, this research confirms that learning should be a coordinated, emotional and enriched experience including physical activity and social relationships. By applying the integrated teaching methods proposed here, the learning difficulties that many pupils have in these subjects can be considerably reduced.

This study shows the successful acquisition of geometric knowledge and skills via the application of a specific PA program, and it is possible that learning content from other subjects via integration with teaching physical education could also be effective.

## Supporting information

S1 Table Data math and PE. (XLSX)
